# Prevalence and β-Lactam Resistance of Enterobacterales Isolated from Urinary Samples in a Regional Hospital in Poland: Special Emphasis on Elderly Population

**DOI:** 10.3390/pathogens15080840

**Published:** 2026-08-12

**Authors:** Łucja Dudzik, Paweł Migdał, Piotr Misiąg, Paweł Krzyżek, Ewa Dworniczek

**Affiliations:** 1 The Provincial Specialist Hospital in Włocławek, 87-800 Włocławek, Poland; lucja.dudzik126@gmail.com; 2 Department of Bees Breeding, Institute of Animal Husbandry, Wroclaw University of Environmental and Life Sciences, 51-630 Wrocław, Poland; pawel.migdal@upwr.edu.pl; 3 Faculty of Medicine, Wroclaw Medical University, 50-345 Wrocław, Poland; piotr.misiag@student.umw.edu.pl; 4 Department of Microbiology, Faculty of Medicine, Wroclaw Medical University, 50-368 Wrocław, Poland

**Keywords:** Enterobacterales, antibiotic resistance, β-lactams, prevalence

## Abstract

Urinary tract infections (UTIs) are among the most common bacterial infections worldwide. The growing prevalence of antimicrobial resistance among Enterobacterales, particularly to β-lactam antibiotics, contributes to treatment failures and prolonged hospital stays. This single-center descriptive surveillance study aimed to evaluate the distribution of Enterobacterales recovered from culture-positive urine samples in the Provincial Specialist Hospital in Włocławek, Poland, and to determine the prevalence of β-lactam resistance phenotypes among these isolates. Urine culture results collected between January and December 2025 from all hospital wards and the outpatient urology clinic were retrospectively analyzed in this study, with 1090 positive urine cultures included. For a subset of 85 isolates, microbiological findings were additionally linked with patients’ clinical characteristics. Bacteria of the genus *Escherichia* were the most frequently isolated (53.8%), followed by *Klebsiella* (22.9%) and *Proteus* (14.2%), with the remaining Enterobacterales genera recovered at frequencies ranging from 0.6% to 4.5%. Most isolates presented no detectable β-lactam resistance phenotype; however, resistance mediated by β-lactamase production was observed in some of them. Phenotypic ESBL and AmpC producers accounted for 25.3% and 0.6% of isolates, respectively, whereas phenotypic carbapenemase-producing strains were detected less frequently, with MBL and KPC producers representing 1.4% and 0.3% of isolates, respectively. No clear associations were identified between patient characteristics and the occurrence of specific bacterial isolates or resistance mechanisms, which may be explained by the relatively homogeneous study population, the majority of whom were ≥70 years old (66/85; 77.6%) and had a similar burden of comorbidities. In conclusion, the current level of β-lactam resistance phenotypes among urinary Enterobacterales in our setting can be considered moderate. Nevertheless, continuous surveillance of local urine-derived bacteria and their antimicrobial resistance profiles remains essential for optimizing effective control strategies.

## 1. Introduction

Urinary tract infections (UTIs) are a major global health concern affecting individuals of all ages and both sexes across diverse patient populations [1]. Approximately 150 million UTI cases are reported worldwide each year, generating more than $6 billion in direct healthcare costs. Although UTIs most commonly affect the lower urinary tract, inadequate treatment may result in serious complications, including pyelonephritis, sepsis, and death. The risk of developing UTIs, as well as experiencing a more severe disease course, is increased by several clinical conditions, including immunosuppression, urinary tract obstruction, diabetes mellitus, and neurological disorders. Furthermore, recurrent UTIs can significantly impair patients’ quality of life and contribute to the growing burden of antimicrobial resistance [2].

It is important to note that the etiology of community-acquired UTIs is predominantly bacterial, accounting for nearly 95% of cases. Although bacteria still predominate in hospital-acquired UTIs, their prevalence is slightly lower [3,4]. Over 50% of these infections are caused by *Escherichia coli*, particularly uropathogenic strains (UPEC), with the remaining cases predominantly caused by other Gram-negative rods, including *Klebsiella pneumoniae* and *Proteus mirabilis* [5,6,7]. These microorganisms are the leading causative agents of uncomplicated UTIs and encode a wide range of virulence factors that enable colonization of the urinary tract and persistence despite highly effective host defense mechanisms [8]. Because UTIs are associated with a substantially increased cost per patient and constitute a significant source of healthcare expenditure [9,10], the need for appropriate and effective treatment is of the utmost importance.

According to the World Health Organization (WHO) Bacterial Priority Pathogens List, third-generation cephalosporin- and carbapenem-resistant Enterobacterales represent a particularly important group of microorganisms [11]. This list precisely specifies *E. coli*, *K. pneumoniae* and species belonging to *Proteus*, *Enterobacter*, *Citrobacter*, *Morganella*, and *Serratia* genera. These pathogens pose a significant threat to human health and can easily acquire resistance to β-lactams, including ESBL (extended-spectrum β-lactamase), AmpC cephalosporinase, KPC (*Klebsiella pneumoniae* carbapenemase), MBL (metallo-β-lactamase), and OXA-48 (oxacillinase-48) phenotypes. ESBL production is one of the most frequently encountered resistance mechanisms among Enterobacterales causing UTIs, conferring resistance primarily to penicillins, most representatives of cephalosporins, and monobactams. AmpC exhibits a similar resistance profile to β-lactams as ESBL does, but also exhibits resistance to old-generation β-lactamase inhibitors. AmpC resistance occurs as an inducible or derepressed chromosomal mechanism, or as a plasmid-mediated one. Notably, exposure to β-lactam antibiotics can induce AmpC overproduction, even in initially susceptible isolates, significantly complicating treatment decisions [12]. By contrast, resistance to carbapenems is associated with the production of carbapenemases, such as KPC, OXA-48, and MBL, leading to the ineffectiveness of virtually all representatives of β-lactams [11,13].

Enteric Gram-negative bacteria readily develop resistance to β-lactams due to efficient horizontal transfer of plasmid-encoded β-lactamase genes, strong antibiotic selection pressure, and biofilm formation during UTIs [14]. Moreover, patients who develop infections caused by pathogens resistant to commonly used antibiotics are characterized by greater healthcare resource utilization, higher treatment costs, and a greater likelihood of progression to complicated UTI compared with patients infected with antibiotic-susceptible strains [15]. Untreated or inadequately treated UTIs may lead to complications, such as pyelonephritis, renal abscess, obstetric complications, including preterm delivery, kidney damage, and, in severe cases, urosepsis [16,17]. The potential progression of urosepsis to septic shock, often requiring hospitalization and intensive care unit treatment, further underscores the global burden of UTIs [18].

In line with the issues outlined above, the aim of the current study was to determine the prevalence of β-lactam resistance phenotypes among Enterobacterales isolated from urine samples in a Regional Hospital in Poland and to establish the association of this phenomenon with patients’ clinical parameters.

## 2. Materials and Methods

### 2.1. Study Population and Data Collection

The analysis included urine culture results from patients hospitalized at The Provincial Specialist Hospital in Włocławek, Poland. This study was approved by the Bioethical Commission of the Wroclaw Medical University (consent no. KB 41/2025) and consisted of two components: a retrospective analysis of microbiological records, including all urinary Enterobacterales isolates identified between 1 January 2025 and 31 December 2025 (*n* = 1090), and a prospective observational cohort comprising adult patients who provided written informed consent to collect additional clinical information (from 19 July 2025 to 28 November 2025; *n* = 85). The prospective cohort represented a subset of the overall study population included in the retrospective microbiological analysis. Importantly, all data in both components were obtained as part of routine clinical care and processed using standard diagnostic procedures; the prospective component differed only in the prospective collection of patient-level clinical data.

For the retrospective cohort, information on the originating hospital unit or outpatient clinic, bacterial species identification, and antimicrobial susceptibility profiles was collected. For the prospective cohort, additional clinical data, including the reason for hospitalization or outpatient treatment, chronic diseases, ongoing pharmacotherapy, and urinalysis results, were obtained. Because complete clinical information was unavailable for the retrospective cohort, symptomatic UTI could not be reliably distinguished from asymptomatic bacteriuria or urinary tract colonization.

In the retrospective dataset, repeated recovery of the same bacterial taxon from the same patient within the same hospital unit was counted once. However, if the same patient was transferred to another hospital unit and the same taxon was recovered again, it was recorded as a separate observation. Therefore, the 1090 records did not represent 1090 unique patients or unique infection episodes, as hospital-wide patient-level deduplication was not possible.

### 2.2. Microbiological Examination

**Figure 1:**
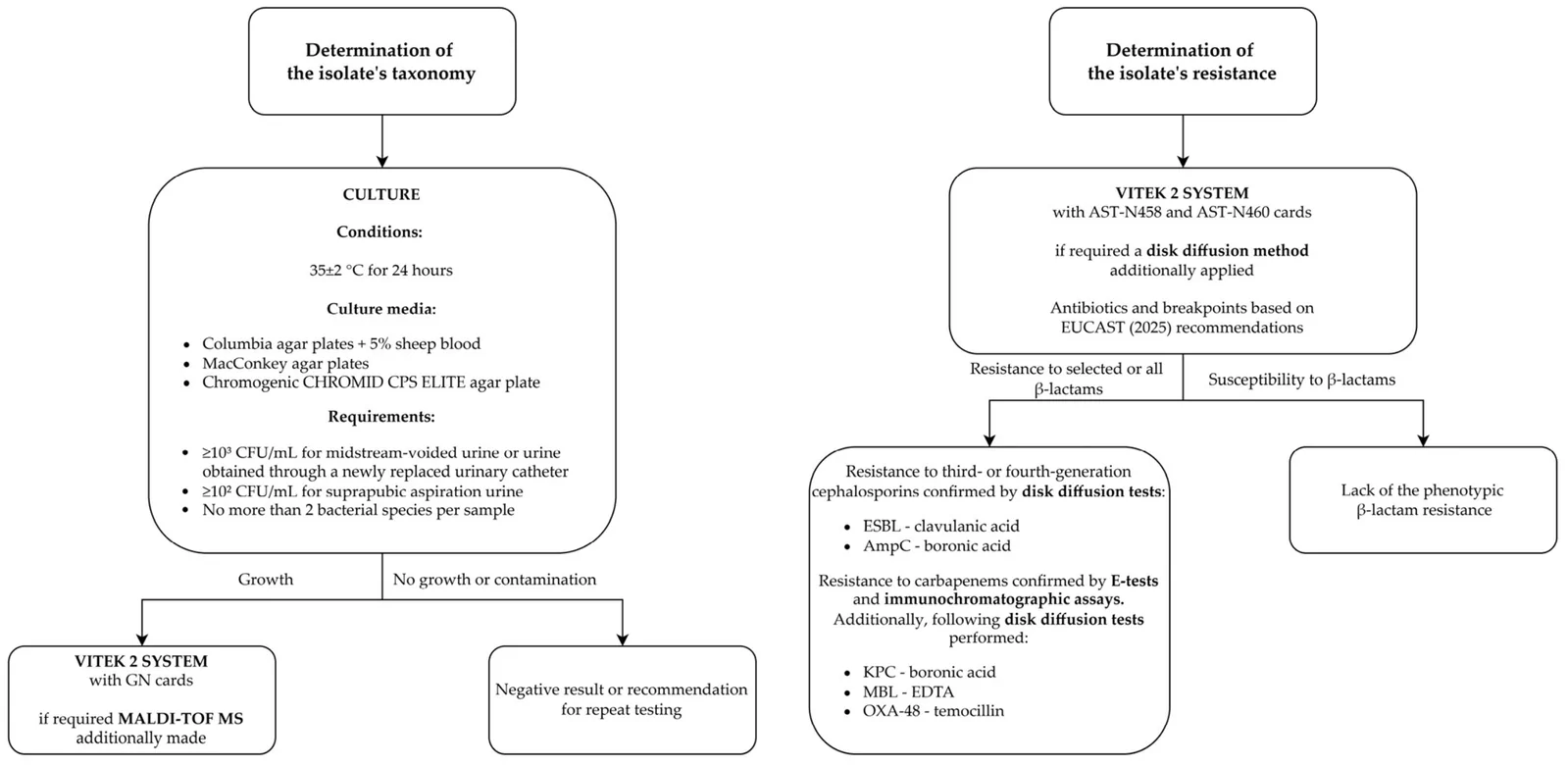
Schematic representation of the microbiological diagnostics of urine samples.

A schematic diagram presenting the identification of taxonomic and β-lactam resistance phenotypes of the isolates is shown in Figure 1. Midstream urine, suprapubic aspiration specimens, and urine from freshly introduced catheters were included. The samples of urine were inoculated on Columbia agar plates with 5% sheep blood (BioMérieux, Marcy-l’Étoile, France) and MacConkey agar plates (BioMérieux, Marcy-l’Étoile, France), and incubated at 35 ± 2 °C for 24 h. Additionally, chromogenic CHROMID CPS ELITE agar plates (BioMérieux, Marcy-l’Étoile, France) were used for the preliminary identification of Enterobacterales. A positive urine culture was defined as bacterial growth of ≥10^2^ CFU/mL for specimens obtained by suprapubic aspiration, and ≥10^3^ CFU/mL for midstream-voided urines or urines obtained through a newly replaced urinary catheter. Additionally, in each case, a positive urine culture was defined as the growth of no more than two bacterial species, with at least one species present at a concentration of ≥10^5^ CFU/mL. Growth of two species that did not meet this criterion, or growth of three or more species, was considered contamination and excluded from the analysis [19]. For the purpose of the current research, only positive cultures containing bacteria from the order Enterobacterales were included in the analysis. If more than one Enterobacterales taxon was recovered from a non-contaminated urine specimen, each isolate was recorded separately.

Following bacterial growth, identification and antimicrobial susceptibility testing were performed using the VITEK 2 system (BioMérieux, Marcy-l’Étoile, France). GN cards (BioMérieux, Marcy-l’Étoile, France) were used for bacterial identification, while AST cards containing different panels of β-lactam antibiotics (including AST-N458 and AST-N460; BioMérieux, Marcy-l’Étoile, France) were used for susceptibility testing. MALDI-TOF MS (Sirius, Bruker Daltonics, Bremen, Germany) was additionally employed in cases where rapid identification was required. Drug susceptibility testing and breakpoint interpretation were performed based on EUCAST recommendations from 2025 [20]. To classify β-lactam resistance phenotypes, categories “S” and “I” were grouped together as susceptible—with category “I” denoting susceptibility with increased exposure, and category “R” classified as resistant. The β-lactam antibiotics tested included ampicillin, amoxicillin/clavulanic acid (intravenous and oral formulations), piperacillin/tazobactam, cephalexin, cefuroxime (intravenous and oral formulations), cefepime, cefotaxime, ertapenem, imipenem, and meropenem, with the specific antibiotics examined depending on the bacterial species identified. The disk diffusion method was applied as a supplementary approach in cases of ambiguous results obtained with the VITEK 2 system, particularly when discrepancies were observed in cephalosporin susceptibility patterns (e.g., resistance to cefepime with simultaneous susceptibility to ceftazidime).

Isolates identified as resistant to β-lactams during screening were further tested to determine the resistance phenotype. Isolates showing reduced susceptibility to third- and/or fourth-generation cephalosporins were considered potential ESBL or AmpC producers. These phenotypes were subsequently confirmed based on the inhibitory effect of clavulanic acid in the case of ESBL and boronic acid in the case of AmpC. Other resistance mechanisms, such as KPC, MBL, and OXA-48, were initially assessed using the VITEK 2 AST-N460 card (BioMérieux, Marcy-l’Étoile, France). In cases where the obtained results exceeded the breakpoint, additional disk diffusion tests were performed to identify the resistance mechanisms. Minimum inhibitory concentrations were determined using E-test gradient strips (BioMérieux, Marcy-l’Étoile, France). In the case of suspected KPC-type mechanisms, boronic acid was used in disk diffusion testing, whereas for suspected OXA-48 production, the MicroZOT test with temocillin (Oxoid, Dardilly, France) was applied. MBL was detected using an EDTA-based test with a carbapenem disk as a comparative control. Positive screening results of carbapenemases were further confirmed using immunochromatographic assays (O.K.N.V.I RESIST-5, Corist Bio-Concept, Gembloux, Belgium).

### 2.3. Statistical Analysis

Statistical analyses were performed and graphs were produced using R version 4.2.3 with RStudio (R Core Team, 2023), using the packages “agricolae”, “dplyr”, and “emmeans”. Continuous variables were initially assessed for normality using the Shapiro–Wilk test, and categorical variables, including bacterial species, hospital ward, sex, comorbidities, and antimicrobial resistance status, were analyzed using Fisher’s exact test. Exact *p*-values are reported whenever possible. Where appropriate, effect size estimates together with their 95% confidence intervals (95% CIs) are provided to facilitate interpretation of the results. All statistical tests were two-sided, and statistical significance was set at α = 0.05. Owing to the relatively limited sample size, particularly for less frequently identified bacterial genera, the statistical analyses should be regarded as exploratory, and non-significant findings should not be interpreted as evidence of the absence of an association.

## 3. Results

Data on 1090 positive urine cultures were collected during the study period [Table 1]. The most frequently recovered species was *Escherichia coli*, accounting for more than half of all analyzed isolates (586/1090; 53.8%), and the next most prevalent were isolates identified as *Klebsiella pneumoniae* (250/1090; 22.9%) and bacteria belonging to the genus *Proteus* (155/1090; 14.2%). Bacteria that are part of the genera *Enterobacter* (49/1090; 4.5%), *Morganella* (22/1090; 2%), *Citrobacter* (21/1090; 1.9%), and *Serratia* (7/1090; 0.6%) were identified considerably less frequently. Among the less frequently recovered Gram-negative fermentative rods, *Proteus mirabilis* was the most prevalent species (141 isolates), followed by *Proteus penneri* (eight isolates), *Proteus vulgaris* (five isolates), and *Proteus hauseri* (one isolate). Among the *Morganella* isolates, *Morganella morganii* subsp. *morganii* (19 isolates) and *Morganella morganii* subsp. *sibonii* (three isolates) were detected. The genus *Citrobacter* comprised *Citrobacter koseri* (10 isolates), *Citrobacter freundii* (seven isolates), *Citrobacter braakii* (two isolates), and *Citrobacter youngae* (two isolates). The *Serratia* isolates encompassed *Serratia marcescens* (five isolates), *Serratia fonticola* (one isolate), and the *Serratia liquefaciens* group (one isolate). The genus *Enterobacter* was represented only by isolates classified as *Enterobacter cloacae* complex (49 isolates).

The majority of isolates did not present any β-lactam resistance phenotypes (790/1090; 72.5%); however, the presence of the ESBL phenotype was also observed in a large proportion of strains (276/1090; 25.3%) [Table 2]. Among *Escherichia* isolates, 82.8% were lacking a β-lactam-resistant phenotype (485/586), whereas the remaining were characterized by the presence of the ESBL resistance mechanism. Isolates of the genus *Proteus* demonstrated a profile similar to that observed for *E. coli*, with a predominance being negative for β-lactam resistance phenotypes (125/155; 80.6%) and a slightly higher proportion of ESBL-positive isolates. A different resistance profile was observed among *Klebsiella* isolates, of which only 39.6% had no detectable β-lactam resistance phenotype (99/250). In this group, ESBL was the predominant resistance mechanism (134/250; 53.6%), while MBL was detected in 6% (15/250) and KPC in 0.8% (2/250) of isolates. A different distribution of resistance mechanisms was observed in *Enterobacter*, where 69.4% of isolates did not present any β-lactam resistance phenotypes (34/49). Among the rest of the isolates of *Enterobacter*, ESBL was the predominant mechanism (9/49; 18.4%), whereas AmpC was detected in 10.2% (5/49) and KPC in 2% (1/49) of them. A similar profile was observed among *Morganella* isolates, of which 86.4% were without a β-lactam resistance phenotype (19/22), while the remaining were characterized by the presence of ESBL or AmpC phenotypes. All *Citrobacter* and *Serratia* isolates were negative for β-lactam resistance phenotypes (21/21 and 7/7, respectively).

The highest number of isolates was obtained from the internal medicine ward (475/1090; 43.6%) [Table 3]. The urology outpatient clinic and the urology ward ranked second (221/1090; 20.3%) and third (153/1090; 14%), respectively. Similar numbers of *Klebsiella* isolates were recovered from the urology outpatient clinic and the urology ward (43/250 and 30/250 isolates, respectively). A comparable distribution was also observed for *Proteus* isolates, with isolates originating from the urology outpatient clinic (40/155) and from the urology ward (25/155). A greater difference was observed for *Escherichia* isolates, with a discrepancy of 30 isolates between the two settings (75/586 vs. 105/586). In the case of *Serratia*, which accounted for only 0.6% of all isolates, strains were recovered from the urology ward, the urology outpatient clinic, and a single isolate from the pulmonology ward. The remaining hospital wards demonstrated greater diversity in the collected isolates.

**Figure 2:**
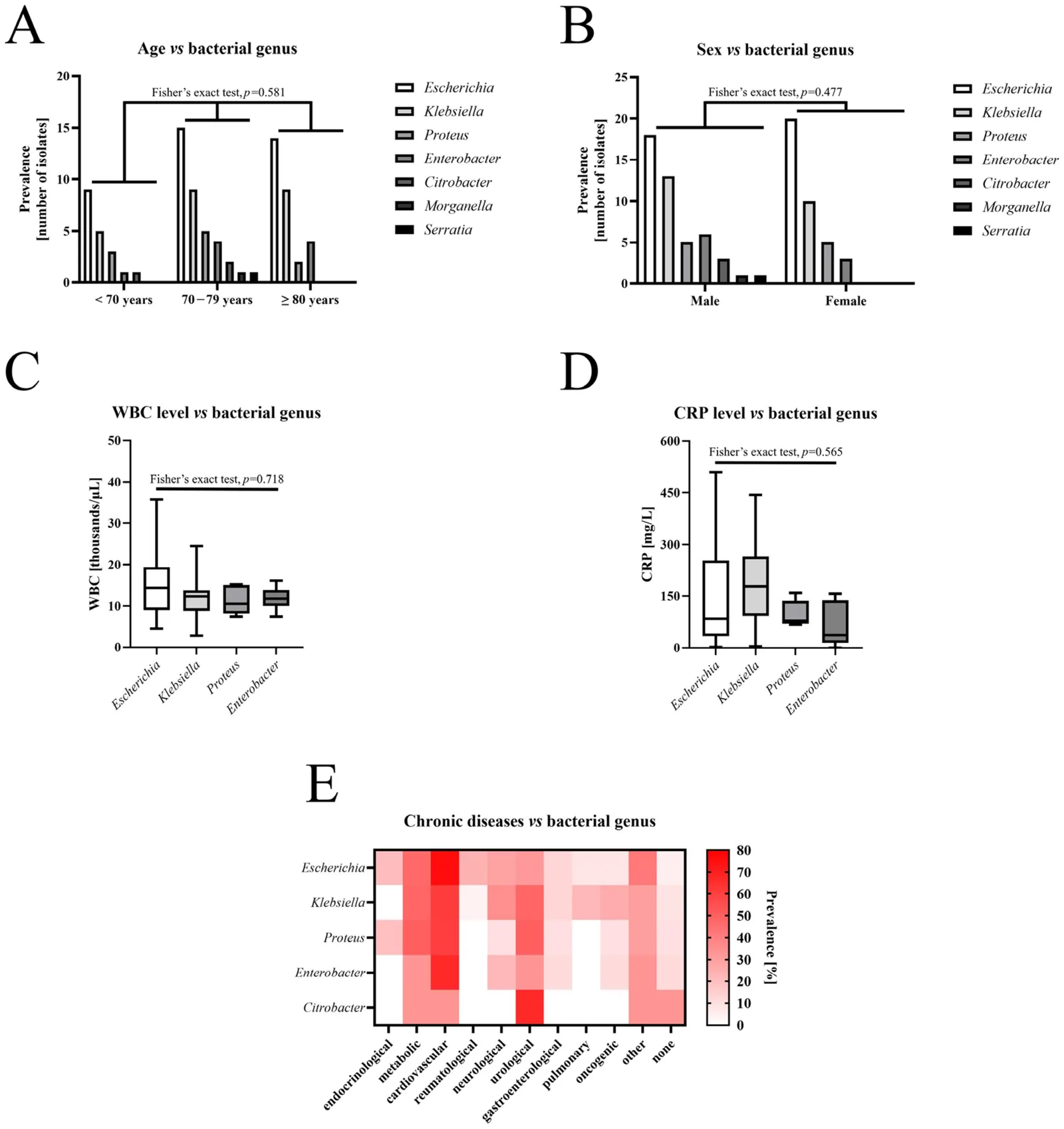
The relationship between the identified urine-borne bacterial isolates and the following patient characteristics: (**A**) age (the mean percentage (±SD) of patients in each age group; *n* = 85), (**B**) sex (the mean percentage (±SD) of patients of each sex; *n* = 85), (**C**,**D**) immunological markers (the median (IQR; min–max) WBC values [white blood cells in thousand/µL] and CRP values [C-reactive protein in mg/L]; *n* = 63), and (**E**) chronic disease status (the heat map showing the mean percentage of patients with each underlying disease, with patients eligible for inclusion in multiple categories; *n* = 83). Detailed data for Panel (**E**), along with statistical analysis values, is provided in Supplementary Table S1. Additional notes: For Panels (**C**,**D**), immunological marker data were available for 64 patients (tests were not performed in the remaining cases). One patient was excluded from the analysis because the corresponding bacterial genus was represented by a single isolate, precluding meaningful comparison at the genus level. Therefore, the final analysis of Panels (**C**,**D**) included 63 patients. For Panel (**E**), two patients were excluded for the same reason.

To obtain a more accurate epidemiological picture, our team determined the relationship between the type of isolated bacteria and the patient parameters in the next stage of research [Figure 2]. In this context, data from 79 patients with 85 isolates were used, with most bacterial isolates recovered from male patients (47/85; 55.3%). All *Citrobacter*, *Morganella*, and *Serratia* isolates originated exclusively from male patients, and *Klebsiella* isolates were predominantly recovered from men, accounting for 57% (13/23) of all isolates. *Proteus* isolates were recovered in equal numbers from women and men (5/10, 50% each). The exception to this phenomenon was *Escherichia*, for which the majority of isolates were obtained from urine samples collected from female patients (53%, 20/38). Most bacterial isolates were obtained from older individuals, particularly those aged 70–79 years, and among *Escherichia* isolates, 37% (15/38) originated from patients within this age group. A similar proportion was observed for *Klebsiella* isolates (39%; 9/23). No *Serratia*, *Morganella*, or *Citrobacter* isolates were obtained from patients older than 79 years. In the case of *Enterobacter*, isolates were distributed equally between patients aged 70–79 years and those aged ≥80 years, with both groups accounting for 44% (4/9) of isolates. The association between inflammation markers and the genus of bacteria was also analyzed. For white blood cell (WBC) counts, the highest median was observed in patients with *Escherichia* isolates (14.3 thousand/µL), whereas the lowest was recorded in patients with *Proteus* isolates (10.5 thousand/µL). Greater variability was observed in the median C-reactive protein (CRP) levels. In the case of CRP values, the highest median was observed in patients with *Klebsiella* isolates (177.5 mg/L), whereas the lowest was recorded in patients with *Enterobacter* isolates (37 mg/L). Equally noteworthy were the associations between comorbidities and the isolated bacterial genera. Most isolates were obtained from urine samples of patients with concomitant cardiovascular diseases. The mean prevalences for *Escherichia* and *Klebsiella* isolates were 76% and 61%, respectively. Regarding endocrine disorders, *Escherichia* and *Proteus* isolates were associated with mean frequencies of 21% and 20%, respectively. Metabolic diseases, including primarily diabetes mellitus, obesity, and lipid disorders, were identified in patients from whom all analyzed bacterial species were isolated. The highest proportion was observed in patients with *Klebsiella* isolates (48%), whereas the lowest proportion was found among patients with *Citrobacter* and *Enterobacter* isolates (33% each). In the case of *Citrobacter*, most isolates were associated with the presence of urological diseases (67%). A relatively large proportion of patients with urological disorders also had *Klebsiella* isolates (48%). Despite all these observed differences in patient parameters, statistical analyses did not demonstrate any significant association between the type of bacterial isolate and the evaluated clinical parameters within the analyzed patient population [Supplementary Table S1]. Because of the relatively small sample size, the statistical analyses should be considered exploratory, and non-significant results should not be interpreted as evidence of the absence of an association.

**Figure 3:**
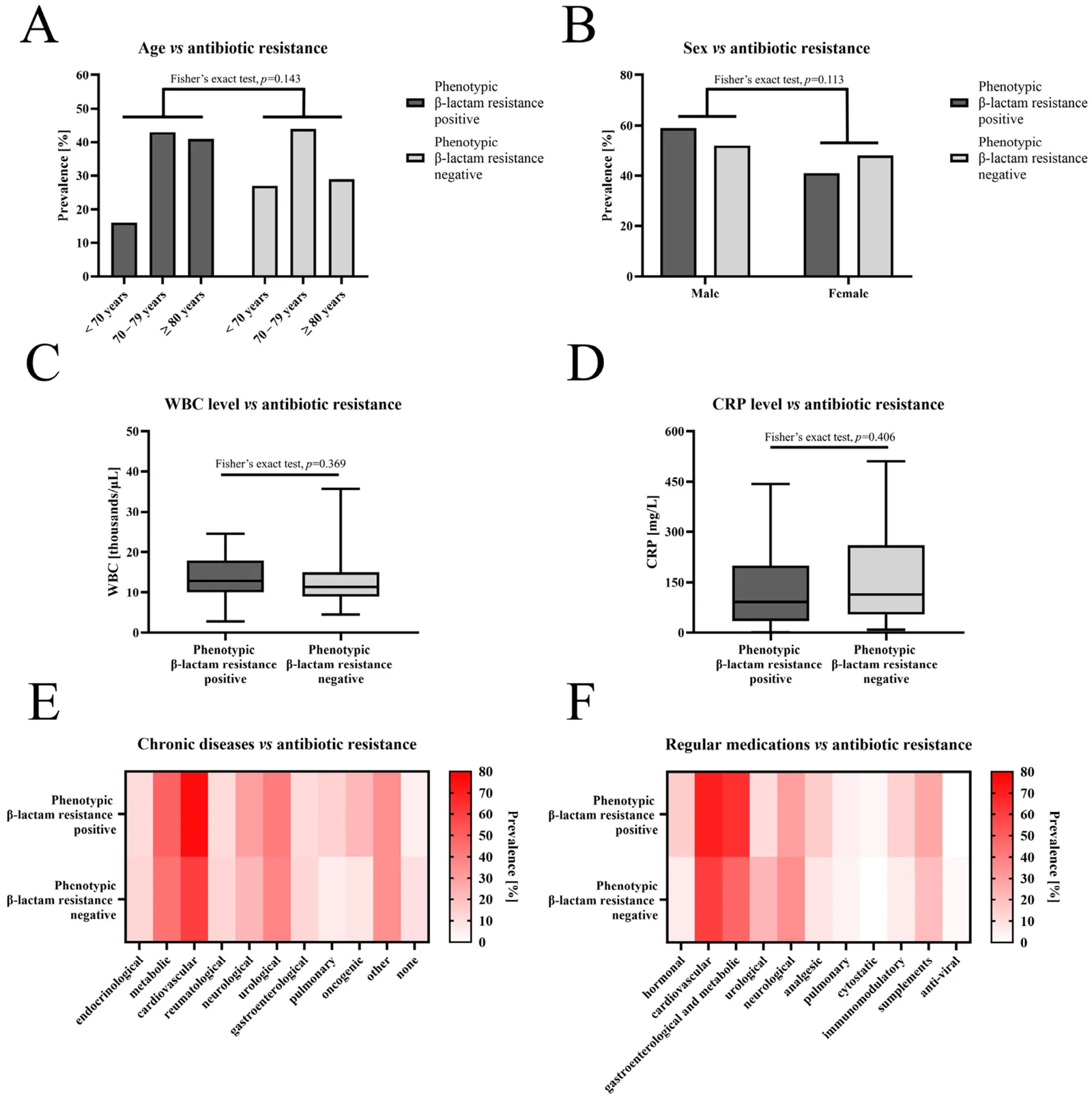
The relationship between the presence of β-lactam resistance phenotypes of the identified urine-borne bacterial isolates and the following patient characteristics: (**A**) age (the mean percentage (±SD) of patients in each age group; *n* = 85), (**B**) sex (the mean percentage (±SD) of patients of each sex; *n* = 85), (**C**,**D**) immunological markers (the median (IQR; min–max) WBC values [white blood cells in thousand/µL] and CRP values [C-reactive protein in mg/L]; *n* = 64), (**E**) chronic disease status (the heat map showing the mean percentage of patients with each underlying disease, with patients eligible for inclusion in multiple categories; *n* = 85), and (**F**) regular medication (the heat map showing the mean percentage of patients using the selected regular medications, with patients eligible for inclusion in multiple categories; *n* = 85). Detailed data for Panels (**E**,**F**), along with statistical analysis values, is provided in Supplementary Table S3. Additional notes: For Panels (**C**,**D**), immunological marker data were available primarily for only 64 patients (tests were not performed in the remaining cases).

Finally, our team aimed to determine the relationship between the presence of β-lactam resistance phenotypes of the isolated bacteria and the patient parameters [Figure 3]. Once again, data coming from 79 patients and a total of 85 isolates were used. Aggregated data showing the susceptibility profile of the analyzed isolates to all tested β-lactams together with β-lactam resistance phenotypes of these isolates are presented in Supplementary Table S2. Since the majority of β-lactam resistance phenotypes were associated exclusively with the ESBL phenotype (30/37; 81.1%), we decided to limit our analysis to the β-lactam resistance-positive vs. -negative phenotype axis. Considering patient sex, 37/85 isolates exhibited β-lactam-resistant phenotypes, of which 59% (22/37) were recovered from males. The mean age of patients from whom isolates with β-lactam-resistant phenotypes were obtained was 77.3 years. The largest group consisted of patients aged between 70 and 79 years, accounting for 43% (16/37) of all these isolates. It is also worth noting that a high proportion of isolates with β-lactam resistance phenotypes was observed among elderly patients, who constituted 41% (15/37) of the analyzed group, whereas among younger patients, this proportion was only 16% (6/37). Considering immunological markers, the median WBC count in patients with isolates presenting β-lactam resistance phenotypes was 12.9 thousand/µL, compared to 11.4 thousand/µL in those with isolates without this phenotype. Patients with isolates lacking a β-lactam-resistant phenotype had a median CRP value of 113 mg/L, whereas in those with isolates positive for this phenotype, the value was 91.3 mg/L. Cardiovascular diseases had the highest frequency among patients with both phenotype variants, the values of which counted for 76% and 60% in β-lactam resistance-positive vs. -negative phenotypes, respectively. Furthermore, metabolic and urological diseases ranked second and third in both groups of patients, respectively. Among patients with isolates with the presence of β-lactam resistance phenotypes, the mean prevalence for metabolic and urological diseases was 49% and 41%, respectively, whereas in those with isolates without this phenotype, the corresponding values were 44% and 38%. Considering the types of medications used by patients participating in this study, cardiovascular drugs constituted the largest group in both patient groups. Among patients with isolates having β-lactam-resistant phenotypes, the use of cytostatic drugs (3%) and immunosuppressive agents (14%) was also observed. As in the previous analysis, despite all these observed differences, statistical analyses did not demonstrate an association between the presence of β-lactam resistance phenotypes and the evaluated patient parameters within our study group [Supplementary Table S3]. Once again, given the relatively small sample size, the statistical analyses should be considered exploratory, and non-significant findings should not be interpreted as evidence of the absence of an association.

## 4. Discussion

Monitoring the local epidemiology of microorganisms with uropathogenic potential is essential for rational antibiotic therapy at both regional and global scales. Additionally, identifying local antimicrobial resistance mechanisms and implementing effective infection control measures provide valuable support in addressing the growing challenge of antimicrobial resistance [5]. In our center, 1090 positive urine cultures were identified in 2025. The distribution of bacteria belonging to the Enterobacterales order was consistent with that reported by other healthcare centers, including multi-profile hospitals in Poland and other European countries. Both our team and others indicate that *Escherichia* and *Klebsiella* were the predominant members of Enterobacterales, highlighting their key role as etiological agents of UTIs [21,22,23,24]. Additionally, similarly to our study, another Polish team also identified *Proteus* as one of the most frequently isolated urine-derived bacteria apart from the two former genera [23]. The detection rate of other Enterobacterales is significantly lower; however, clinicians emphasize the need for continuous epidemiological surveillance of these microorganisms [25].

Given the increasing prevalence of antimicrobial resistance among bacteria, systematic monitoring of antimicrobial susceptibility patterns is essential for detecting emerging resistance trends and adjusting therapies [26,27]. In our research, ESBL-producing variants accounted for 17.2% (101/586) of *Escherichia* isolates and 53.6% (134/250) of *Klebsiella* isolates. This prevalence is within the range reported in recent UTI surveillance studies, which have documented ESBL rates of approximately 24–38% among *Escherichia* and 39–46% among *Klebsiella* urinary isolates in tertiary-care settings [28]. Additionally, the recent Silesian multicenter study reported ESBL prevalence among Enterobacterales of up to 32.9% in hospitalized patients, suggesting similarly to us a substantial reservoir of resistant microorganisms in Polish hospitals [29]. Moreover, in our study, isolates producing other β-lactamases, including AmpC, KPC, and MBL, were also identified. Phenotypic AmpC was detected primarily among *Enterobacter* isolates (10.2%), being consistent with previous reports indicating that this genus has the greatest potential for inducible AmpC β-lactamase production among Enterobacterales [12]. Although KPC- and MBL-producing isolates accounted for only a small proportion of the analyzed isolates, their detection is of considerable epidemiological and clinical importance, as these phenotypes are associated with resistance to the most important classes of antibiotics used in the treatment of severe infections caused by Enterobacterales [30]. Therefore, further monitoring of this phenomenon is highly important.

In the present study, no significant associations were found between the analyzed patient parameters and the occurrence of specific bacterial genera/species or the presence of β-lactam resistance phenotypes. This may be attributed to the characteristics of the study population, which was relatively homogeneous in terms of age and comorbidities. A substantial proportion of our patients were aged ≥70 years (66/85; 77.6%). In such a population, multimorbidity and the presence of multiple age-related risk factors may diminish the influence of individual clinical parameters on the type of isolated microorganism and its antimicrobial resistance profile [31,32,33]. Previous research in elderly populations has similarly reported that major comorbid conditions do not necessarily translate into substantial differences in distribution or resistance profiles of bacteria isolates from the urinary tract [34]. Additionally, a recent study by Ming et al. highlighted the limited diagnostic reliability of conventional urinary inflammatory markers (such as pyuria and leukocyte esterase) in elderly patients, as many of them exhibit a diminished inflammatory response [35]. In line with the issues mentioned above, in another study, it was concluded that the occurrence of healthcare-associated infections in elderly hospitalized patients was influenced by a complex interplay of age-related immune dysfunction, nutritional status, and comorbidity burden, while individual clinical variables often had limited predictive value when analyzed separately [36].

Several limitations of the present study should be acknowledged. First, the detection of β-lactam resistance mechanisms was based solely on phenotypic methods and was not confirmed by molecular analyses. Although genetic characterization would have provided definitive evidence for the presence or absence of specific resistance mechanisms, such data were not available from the hospital’s epidemiological records. Secondly, the microbiology laboratory processing the urine samples had access only to the microbiological culture and antimicrobial susceptibility results and did not have access to comprehensive clinical information, including urinary tract symptoms, the presence of leukocyturia, or decisions regarding antibiotic treatment. Consequently, in the retrospective component of the study, it was not possible to reliably distinguish symptomatic UTIs from asymptomatic bacteriuria or urinary tract colonization. Thirdly, the analysis was conducted in a single center and included a relatively limited number of isolates, both of which may restrict the generalizability of the findings. On the other hand, most published studies focus on large tertiary-care or university hospitals, whereas data regarding the epidemiology in smaller healthcare facilities, characterized by a lower patient throughput and microbial samples, remain scarce. Despite these limitations, we believe that the present findings provide valuable insights into the local epidemiology of bacteria isolated from the urinary tract in Poland and may contribute to national surveillance efforts aimed at monitoring the prevalence and antimicrobial resistance patterns of Enterobacterales with uropathogenic potential.

As a single-center descriptive surveillance study, our findings provide a local epidemiological perspective on the distribution of urinary Enterobacterales and their β-lactam resistance phenotypes. The presence of β-lactam resistance phenotypes among Enterobacterales recovered from urine samples in our setting remains at a moderate level. Nevertheless, the notable prevalence of ESBL-producing isolates warrants particular attention. Continuous surveillance of local urinary bacteria and their resistance profiles is essential to optimize empirical antimicrobial treatment and to support evidence-based prevention and control efforts.

## Figures and Tables

**Table 1:** Annual prevalence of bacterial isolates recovered from urine samples.

Classification of the Isolates		Number of Isolates	
Genus	Species		
*Escherichia*	*Escherichia coli*		*n* = 586 (53.8%)
*Klebsiella*	*Klebsiella pneumoniae*		*n* = 250 (22.9%)
*Proteus*	*Proteus mirabilis*	*n* = 141	*n* = 155 (14.2%)
	*Proteus penneri*	*n* = 8	
	*Proteus vulgaris*	*n* = 5	
	*Proteus hauseri*	*n* = 1	
*Enterobacter*	*Enterobacter cloacae* complex		*n* = 49 (4.5%)
*Morganella*	*Morganella morganii* subsp. *morganii*	*n* = 19	*n* = 22 (2%)
	*Morganella morganii* subsp. *sibonii*	*n* = 3	
*Citrobacter*	*Citrobacter koseri*	*n* = 10	*n* = 21 (1.9%)
	*Citrobacter freundii*	*n* = 7	
	*Citrobacter braakii*	*n* = 2	
	*Citrobacter youngae*	*n* = 2	
*Serratia*	*Serratia marcescens*	*n* = 5	*n* = 7 (0.6%)
	*Serratia fonticola*	*n* = 1	
	*Serratia liquefaciens*	*n* = 1	
**Total**			***n*** ** = 1090 (100%)**

**Table 2:** Annual distribution of β-lactam resistance phenotypes in bacterial isolates recovered from urine samples.

β-lactam Resistance Phenotype	Bacterial Genus							
	*Escherichia*	*Klebsiella*	*Proteus*	*Enterobacter*	*Morganella*	*Citrobacter*	*Serratia*	All
Negative *	485 (82.8%)	99 (39.6%)	125 (80.6%)	34 (69.4%)	19 (86.4%)	21 (100%)	7 (100%)	790 (72.5%)
ESBL	101 (17.2%)	134 (53.6%)	30 (19.4%)	9 (18.4%)	2 (9.1%)	0 (0%)	0 (0%)	276 (25.3%)
AmpC	0 (0%)	0 (0%)	0 (0%)	5 (10.2%)	1 (4.5%)	0 (0%)	0 (0%)	6 (0.6%)
KPC	0 (0%)	2 (0.8%)	0 (0%)	1 (2%)	0 (0%)	0 (0%)	0 (0%)	3 (0.3%)
MBL	0 (0%)	15 (6%)	0 (0%)	0 (0%)	0 (0%)	0 (0%)	0 (0%)	15 (1.4%)
**Total**	**586 (100%)**	**250 (100%)**	**155 (100%)**	**49 (100%)**	**22 (100%)**	**21 (100%)**	**7 (100%)**	**1090 (100%)**

* Note: A negative β-lactam resistance phenotype indicates that no phenotypic β-lactam resistance mechanism was detected. Resistance to antimicrobial agents other than β-lactams was not assessed in this study and therefore cannot be inferred from these results.

**Table 3:** (**A**) Annual distribution of bacterial isolates recovered from urine samples of patients across different hospital units, along with (**B**) a statistical analysis of these data.

(**A**)								
**Sampling Unit**	**Bacterial Genus**							
	***Escherichia***	***Klebsiella***	***Proteus***	***Enterobacter***	***Morganella***	***Citrobacter***	***Serratia***	**All**
Internal medicine	263 (44.9%)	117 (46.8%)	66 (42.6%)	15 (30.6%)	6 (27.3%)	8 (38.1%)	0 (0%)	475 (43.6%)
Urological outpatient clinic	105 (17.9%)	43 (17.2%)	40 (25.8%)	15 (30.6%)	6 (27.3%)	9 (42.9%)	3 (42.9%)	221 (20.3%)
Urology	75 (12.8%)	30 (12%)	25 (16.1%)	11 (22.4%)	6 (27.3%)	3 (14.3%)	3 (42.9%)	153 (14%)
Pediatrics	45 (7.7%)	2 (0.8%)	4 (2.6%)	2 (4.1%)	0 (0%)	0 (0%)	0 (0%)	53 (4.9%)
Neurology	26 (4.4%)	7 (2.8%)	6 (3.9%)	0 (0%)	3 (13.6%)	0 (0%)	0 (0%)	42 (3.9%)
Intensive care unit	6 (1%)	30 (12%)	2 (1.3%)	2 (4.1%)	0 (0%)	0 (0%)	0 (0%)	40 (3.7%)
Pulmonology	14 (2.4%)	4 (1.6%)	4 (2.6%)	1 (2%)	0 (0%)	0 (0%)	1 (14.3%)	24 (2.2%)
General surgery	12 (2%)	8 (3.2%)	3 (1.9%)	1 (2%)	0 (0%)	0 (0%)	0 (0%)	24 (2.2%)
Gynecology	16 (2.7%)	1 (0.4%)	1 (0.6%)	0 (0%)	0 (0%)	1 (4.8%)	0 (0%)	19 (1.7%)
Neurosurgery	6 (1%)	2 (0.8%)	1 (0.6%)	2 (4.1%)	1 (4.5%)	0 (0%)	0 (0%)	12 (1.1%)
Cardiology	3 (0.5%)	5 (2%)	1 (0.6%)	0 (0%)	0 (0%)	0 (0%)	0 (0%)	9 (0.8%)
Orthopedics	6 (1%)	0 (0%)	2 (1.3%)	0 (0%)	0 (0%)	0 (0%)	0 (0%)	8 (0.7%)
Dermatology	7 (1.2%)	0 (0%)	0 (0%)	0 (0%)	0 (0%)	0 (0%)	0 (0%)	7 (0.6%)
Neonatology	2 (0.3%)	0 (0%)	0 (0%)	0 (0%)	0 (0%)	0 (0%)	0 (0%)	2 (0.2%)
Otolaryngology	0 (0%)	1 (0.4%)	0 (0%)	0 (0%)	0 (0%)	0 (0%)	0 (0%)	1 (0.1%)
**Total**	**586 (100%)**	**250 (100%)**	**155 (100%)**	**49 (100%)**	**22 (100%)**	**21 (100%)**	**7 (100%)**	**1090 (100%)**
(**B**)								
**Bacterial Genus**			**Fisher’s Exact Test Analysis**					
			***p*** **-Value**		**OR**		**95% CI**	
*Escherichia*			0.012		2.24		1.18–5.71	
*Klebsiella*			0.004		3.14		2.22–3.61	
*Proteus*			0.022		2.34		1.18–6.84	
*Enterobacter*			0.039		2.48		2.25–7.92	
*Morganella*			0.027		2.29		1.38–4.71	
*Citrobacter*			0.015		3.21		2.06–4.78	
*Serratia*			NA *		NA *		NA *	

* OR, 95% CI, and *p*-value could not be reliably estimated because of the limited number of isolates.

## Data Availability

The original contributions presented in this study are included in the article/Supplementary Material. Further inquiries can be directed to the corresponding authors.

## Supplementary Materials

The following supporting information can be downloaded at: https://www.mdpi.com/article/10.3390/pathogens15080840/s1, Table S1: Detailed data regarding the relationship between the isolated urinary bacteria and patient parameters; Table S2: Detailed data regarding β-lactam resistance phenotypes and susceptibility to each of the tested β-lactams for the isolated urinary bacteria; Table S3: Detailed data regarding the relationship between the presence of β-lactam resistance phenotypes of isolated urinary bacteria and patient parameters.

## References

[1] Pietropaolo A. (2023). Urinary Tract Infections: Prevention, Diagnosis, and Treatment. J. Clin. Med..

[2] Stamm W.E., Norrby S.R. (2001). Urinary Tract Infections: Disease Panorama and Challenges. J. Infect. Dis..

[3] Mancuso G., Midiri A., Gerace E., Marra M., Zummo S., Biondo C. (2023). Urinary Tract Infections: The Current Scenario and Future Prospects. Pathogens.

[4] Dudzik Ł., Krzyżek P., Dworniczek E. (2025). A Review on the Current and Future State of Urinary Tract Infection Diagnostics. Int. J. Mol. Sci..

[5] Flores-Mireles A.L., Walker J.N., Caparon M., Hultgren S.J. (2015). Urinary Tract Infections: Epidemiology, Mechanisms of Infection and Treatment Options. Nat. Rev. Microbiol..

[6] Govindarajan D.K., Kandaswamy K. (2022). Virulence Factors of Uropathogens and Their Role in Host Pathogen Interactions. Cell Surf..

[7] McLellan L.K., Hunstad D.A. (2016). Urinary Tract Infection: Pathogenesis and Outlook. Trends Mol. Med..

[8] Bien J., Sokolova O., Bozko P. (2012). Role of Uropathogenic *Escherichia coli* Virulence Factors in Development of Urinary Tract Infection and Kidney Damage. Int. J. Nephrol..

[9] Sulham K., Hammelman E. (2021). 1416. Medicare Spending on Urinary Tract Infections: A Retrospective Database Analysis. Open Forum Infect. Dis..

[10] Murray C.J.L., Ikuta K.S., Sharara F., Swetschinski L., Robles Aguilar G., Gray A., Han C., Bisignano C., Rao P., Wool E. (2022). Global Burden of Bacterial Antimicrobial Resistance in 2019: A Systematic Analysis. Lancet.

[11] World Health Organization (2024). WHO Bacterial Priority Pathogens List, 2024: Bacterial Pathogens of Public Health Importance to Guide Research, Development and Strategies to Prevent and Control Antimicrobial Resistance.

[12] Tamma P.D., Doi Y., Bonomo R.A., Johnson J.K., Simner P.J., Tamma P.D., Doi Y., Bonomo R.A. (2019). A Primer on AmpC β-Lactamases: Necessary Knowledge for an Increasingly Multidrug-Resistant World. Clin. Infect. Dis..

[13] Sivarajan V., Ganesh A.V., Subramani P., Ganesapandi P., Sivanandan R.N., Prakash S., Manikandan N., Dharmarajan A., Arfuso F., Warrier S. (2025). Prevalence and Genomic Insights of Carbapenem Resistant and ESBL Producing Multidrug Resistant *Escherichia coli* in Urinary Tract Infections. Sci. Rep..

[14] Shao F., Li D., Wang J., Tuo Z., Wang Z., Wei W., Wu R., Feng D. (2025). Beta-Lactamase-Mediated Antibiotic Resistance in Urinary Tract Infections: Mechanisms and Therapeutic Strategies. Urogenit. Tract Infect..

[15] Shafrin J., Marijam A., Joshi A.V., Mitrani-Gold F.S., Everson K., Tuly R., Rosenquist P., Gillam M., Ruiz M.E. (2022). Economic Burden of Antibiotic-Not-Susceptible Isolates in Uncomplicated Urinary Tract Infection: Analysis of a US Integrated Delivery Network Database. Antimicrob. Resist. Infect. Control.

[16] Stangl F., Wagenlehner F., Schneidewind L., Kranz J. (2024). Urosepsis: Pathophysiologie, Diagnose Und Management—Ein Update. Die Urol..

[17] Wagenlehner F., Caballero V.R., Maheshwari V., Biswas A., Saini P., Quevedo J., Polifka J., Ruiz L., Cure S. (2025). Efficacy of Treatment Options for Complicated Urinary Tract Infections Including Acute Pyelonephritis: A Systematic Literature Review and Network Meta-Analysis. J. Comp. Eff. Res..

[18] Montelin H., Camporeale A., Hallgren A., Angelin M., Hogvall J., Östholm Balkhed Å., Vading M., Giske C.G., Tängdén T., Angelin M. (2024). Treatment, Outcomes and Characterization of Pathogens in Urinary Tract Infections Caused by ESBL-Producing Enterobacterales: A Prospective Multicentre Study. J. Antimicrob. Chemother..

[19] Bilsen M.P., Jongeneel R.M.H., Schneeberger C., Platteel T.N., van Nieuwkoop C., Mody L., Caterino J.M., Geerlings S.E., Köves B., Wagenlehner F. (2023). Definitions of Urinary Tract Infection in Current Research: A Systematic Review. Open Forum Infect. Dis..

[20] The European Committee on Antimicrobial Susceptibility Testing (EUCAST) (2025). The Breakpoint Tables for Interpretation of MICs and Zone Diameters, Version 15.0.

[21] Futoma-Kołoch B., Sarowska J., Abd El-Salam M., Miñana-Galbis D., Drabová B., Guz-Regner K., Wiśniewska P., Kryniewska V. (2025). Current Insights into Antibiotic Resistance in Uropathogenic *Escherichia coli* and Interventions Using Selected Bioactive Phytochemicals. Antibiotics.

[22] Trześniewska-Ofiara Z., Mendrycka M., Woźniak-Kosek A. (2025). Drug Susceptibility of Uropathogens Isolated from Patients Treated at the Mazovian Specialized Hospital in Radom. Acta Biochim. Pol..

[23] Kawalec A., Józefiak J., Kiliś-Pstrusińska K. (2023). Urinary Tract Infection and Antimicrobial Resistance Patterns: 5-Year Experience in a Tertiary Pediatric Nephrology Center in the Southwestern Region of Poland. Antibiotics.

[24] Petca R.-C., Mareș C., Petca A., Negoiță S., Popescu R.-I., Boț M., Barabás E., Chibelean C.B. (2020). Spectrum and Antibiotic Resistance of Uropathogens in Romanian Females. Antibiotics.

[25] Gajdács M., Urbán E. (2019). Comparative Epidemiology and Resistance Trends of Proteae in Urinary Tract Infections of Inpatients and Outpatients: A 10-Year Retrospective Study. Antibiotics.

[26] Chin T.L., McNulty C., Beck C., MacGowan A. (2016). Antimicrobial Resistance Surveillance in Urinary Tract Infections in Primary Care. J. Antimicrob. Chemother..

[27] Simoni A., Schwartz L., Junquera G.Y., Ching C.B., Spencer J.D. (2024). Current and Emerging Strategies to Curb Antibiotic-Resistant Urinary Tract Infections. Nat. Rev. Urol..

[28] Jo S.B., Ahn S.T., Joo H.J., Kim J.W., Oh M.M. (2025). Carbapenem Resistance and ESBL-Producing Enterobacteriaceae in Patients with Urological Infections from 2012 to 2021 in Three Korean Hospitals. Diagnostics.

[29] Gajda M., Kapturkiewicz C., Kapusta D., Hareza D., Pomorska-Wesołowska M., Kawałek A., Wójkowska-Mach J. (2026). Applying International Urinary Tract Infection Guidelines in Poland: Epidemiologic Insights from Southern Poland. Pol. Arch. Intern. Med..

[30] De Rosa F.G., Corcione S., Cavallo R., Di Perri G., Bassetti M. (2015). Critical Issues for *Klebsiella pneumoniae* KPC-Carbapenemase Producing *K. pneumoniae* Infections: A Critical Agenda. Future Microbiol..

[31] Rodriguez-Mañas L. (2020). Urinary Tract Infections in the Elderly: A Review of Disease Characteristics and Current Treatment Options. Drugs Context.

[32] Gajdács M., Ábrók M., Lázár A., Burián K. (2021). Urinary Tract Infections in Elderly Patients: A 10-Year Study on Their Epidemiology and Antibiotic Resistance Based on the WHO Access, Watch, Reserve (AWaRe) Classification. Antibiotics.

[33] Kocur S.B., Noppenberg M., Sowińska I., Gniadek A. (2023). Selected Risk Factors for Urinary Tract Infections. Probl. Pielęg..

[34] Bonadio M., Costarelli S., Morelli G., Tartaglia T. (2006). The Influence of Diabetes Mellitus on the Spectrum of Uropathogens and the Antimicrobial Resistance in Elderly Adult Patients with Urinary Tract Infection. BMC Infect. Dis..

[35] Ming H., Na W., Tingting H., Huiyi W., Jiaping W. (2026). Low-Inflammatory Response Phenotypes in Elderly Urinary Tract Infections: Pathogen Type, Antimicrobial Resistance, and Comorbidity-Associated Risk Profiles. BMC Geriatr..

[36] Laurent M., Bastuji-Garin S., Plonquet A., Bories P.N., Le Thuaut A., Audureau E., Lang P.O., Nakib S., Liuu E., Canoui-Poitrine F. (2015). Interrelations of Immunological Parameters, Nutrition, and Healthcare-Associated Infections: Prospective Study in Elderly in-Patients. Clin. Nutr..

